# Cell-Type-Specific Length and Cytosolic pH Response of Superficial Cells of *Arabidopsis* Root to Chronic Salinity

**DOI:** 10.3390/plants11243532

**Published:** 2022-12-15

**Authors:** Maria Ageyeva, Alexander Veselov, Vladimir Vodeneev, Anna Brilkina

**Affiliations:** 1 Department of Biochemistry and Biotechnology, National Research Lobachevsky State University of Nizhny Novgorod, 23 Gagarin Avenue, Nizhny Novgorod 603950, Russia; 2 Department of Biophysics, National Research Lobachevsky State University of Nizhny Novgorod, 23 Gagarin Avenue, Nizhny Novgorod 603950, Russia

**Keywords:** salinity, root, root cap, epidermal cells, differentiation zone, pH, pH indicator, Pt-GFP, *Arabidopsis thaliana* L.

## Abstract

Soil salinity negatively affects the growth, development and yield of plants. Acidification of the cytosol in cells of glycophytes was reported under salinity, while various types of plant cells can have a specific reaction under the same conditions. Transgenic *Arabidopsis* plants expressing the pH sensor Pt-GFP in the cytosol were used in this work for determination of morphometric changes and cytosolic pH changes in the superficial cells of *Arabidopsis* roots under chronic salinity in vitro. We did not find changes in the length of the root cap cells, while there was a decrease in the length of the differentiation zone under 50, 75 mM NaCl and the size of the epidermal cells of the differentiation zone under 75 mM NaCl. The most significant changes of cytosolic pH to chronic salinity was noted in columella (decrease by 1 pH unit at 75 mM NaCl) and epidermal cells of the differentiation zone (decrease by 0.6 and 0.4 pH units at 50 and 75 mM NaCl, respectively). In developed lateral root cap cells, acidification of cytosol by 0.4 units occurred only under 75 mM NaCl in the medium. In poorly differentiated lateral cells of the root cap, there were no changes in pH under chronic salinity.

## 1. Introduction

Soil salinity is a major global issue of modern agriculture [1,2]. This problem affects more than 20% of the global irrigated area leading to serious crop losses [3]. The area of salinized soils is expanding at a rate of 1–2 M ha year^−1^ [2,4], and in the next decades, this problem will get worse by climate change [5].

An excess of salts in soils leads to a decrease in the growth and development of the most crops and, ultimately, to decrease in yield [6,7,8,9,10]. An increased salt content in soils have a direct effect on the superficial cells of plant roots including cells of the root cap and the epidermal cells of the differentiation zone [11]. Salt stress is mainly caused by toxic concentrations of sodium (Na^+^) and chloride ions (Cl^−^) in the soil solution, and their negative effects on plants include primary stresses such as osmotic stress and ion imbalance and secondary stresses such as oxidative stress and metabolic changes [11,12,13]. Salinity often leads to a change of intracellular pH to control the Na^+^ and Cl^−^ concentrations in the cell [9,14]. However, the effect of salinity on intracellular pH of various types of cells and tissues requires a detailed study to understand the mechanisms of plant adaptive responses and the possibility of controlling salinity tolerance in plants.

A specific value of pH in the cell is a key condition for the functioning of all living organisms including plants. The H^+^ concentration affects the structure of proteins, the activity of enzymes and ion channels, the carrying capacity of membranes, and, ultimately, the physiological processes of plants, such as photosynthesis and respiration, plant development and growth [15,16,17]. A constant value of cytosolic pH are stabilized by work of buffer systems, H^+^ transporters and organic acids [18]. 

Intercellular and extracellular pHs have a wide regulatory role, so much attention is paid to the study of the pH of the cytoplasm and apoplast of plants. The pH values of various cell organelles of plant protoplasts were determined [17]. Most of the works were carried out on the roots of *Arabidopsis* plants [19,20,21,22,23]. Some studies of cytosolic pH of root cells were carried out on plants of corn [24], radish and mustard [25]. At the same time, there are only few studies that would measure the cytosolic pH of different root parts. In most works, the results of determining the pH of the cytosol of the cells of the root tip, including the elongation zone and the root cap, are presented. The cytosolic pH of these cells is approximately 7.2 [19,20,23]. On the other hand, the study of Martainaire et al. [26] showed an alkalization of cytosolic pH in the differentiation root zone from the epidermal cells deep into the stele. Schulte et al. [21] reported that the pH of the cytoplasm in the cells of the elongation zone near the root tip is 7.4–7.6 and differs from the pH of the cytoplasm of cells in the zone of root hairs near the hypocotyl—7.2–7.3. The pH difference of the apoplast along *Arabidopsis* root was similarly noted [22,23]. The apoplastic pH value of the distal part of the root elongation zone was 4.8, and the apoplastic pH of the root differential zone is 6.0 [22,23]. The pH values of the cytosol and apoplast are interrelated because alkalization of one leads to acidification of the other. Therefore, it can be assumed that there is heterogeneity of the pH of the cytosol of various zones of the root.

Environmental factors affect cytosolic pH [15]. Changes of cytosolic pH of root cells and leaf cells of plants were shown under hypoxia or anoxia [15,21], various light intensities [15], attack of pathogenic microorganisms [27], low or high temperature [28,29] and gravistimulation [23]. Using *Arabidopsis* and pea glycophytes, it was shown that the cytosolic pH of root cells decreases under conditions of elevated NaCl concentrations [14,21,30], while in quinoa halophyte, it increases [14].

In most studies, pH value changes are determined locally for any particular root zone. Considering that each root zone consists of cells different in morphology and functions, it can be assumed that the response of these zones may be different. Similar studies were carried out for the root of *Arabidopsis* upon gravistimulation. Alkalinization of the cytoplasm of columella cells was shown, while no pH changes of cytosol were found elsewhere in the root [23]. 

Various methods such as H^+^-selective microelectrodes, pH-sensitive fluorescent dyes, genetically encoded pH sensors are used to estimate pH in plant cells. For over than 15 years, researchers have been using genetically encoded pH sensors based on green fluorescent protein (GFP) to study the intracellular pH of plant cells [17,21,26,29,31,32,33]. These GFP sensors have pH-sensitive chromophores [29,31], and they can be targeted to various cellular organelles [17]. The use of plants expressing fluorescent proteins makes it possible to describe cell morphology and determine cell and tissue types without histological staining. The constant expression of GFP in plants allows for dynamic studies in vivo.

In this work, the inhibitory effect of chronic salinity on the length of the plant root and its individual zones was reported, when *Arabidopsis* was grown on a nutrient medium with an increased concentration of NaCl. The values of cytosolic pH of epidermal cells of the differentiation zone and various cells of the root cap were determined under normal conditions and under chronic salinity. We show that chronic salinity leads to acidification of the cytosol of lateral root cap tip cells, differentiated lateral root cap cells and epidermal cells of the differentiation zone. 

## 2. Results

### 2.1. Localization of Pt-Gfp in Arabidopsis Root Cells

**Figure 1:**
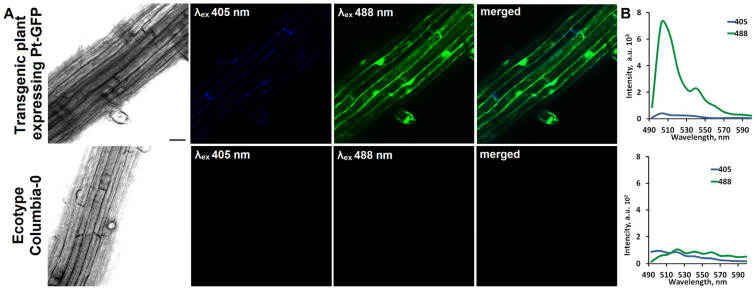
Fluorescence of root cells of *Arabidopsis* transgenic plant expressing Pt-GFP and *Arabidopsis* plant ecotype Columbia. (**A**) Confocal images of root cells. Transmitted light images, fluorescent images (λem 500–550 nm) with excitation at λex 405 nm and λex 488 nm, and images with merged fluorescent channels. Scale bar, 20 μm. (**B**) Spectra of fluorescence of root cells of *Arabidopsis* transgenic plant and *Arabidopsis* plant ecotype Columbia with excitation at λex 405 nm and λex 488 nm.

The effect of salinity on the length and pH of *Arabidopsis* root cells was studied in this work. For this, we used plants expressing the genetically encoded pH indicator Pt-GFP [21]. This ratiometric indicator has three fluorescence excitation peaks, the intensity of which depends on pH. At acidic pH values, all fluorescence excitation peaks at 390 nm, 475 nm and 502 nm are well pronounced. Under alkaline conditions the two peaks at 475 nm and 502 nm is well pronounced. We detected a fluorescent signal along the cell periphery and presumably in the nucleus region of *Arabidopsis* root cells (Figure 1A). This signal is characteristic of Pt-GFP. The fluorescence intensity of root cells of transgenic plants is significantly higher than root cells of nontransgenic *Arabidopsis* plants of the Columbia ecotype. The fluorescence spectrum of the root cells of the transgenic plant clearly shows a fluorescence maximum at 508 nm, which is characteristic of Pt-GFP (Figure 1B). In addition, the spectrum also shows a peak at 540 nm, which is characteristic of all GFP proteins. Both fluorescence peaks are detected in the spectra at λex 488 and λex 405 nm. The fluorescence spectra of the root cells of nontransgenic *Arabidopsis* obtained under similar conditions did not reveal such peaks, and the summary fluorescence level was significantly lower.

**Figure 2:**
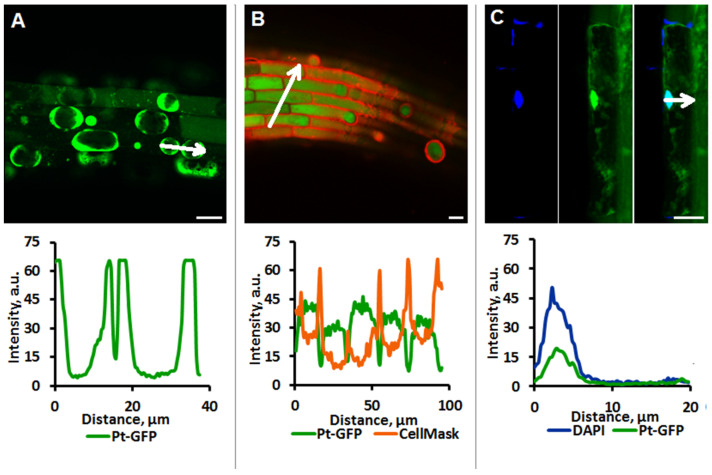
Analysis of Pt-GFP localization in the epidermal root cells of *Arabidopsis* transgenic plants expressing Pt-GFP. (**A**) Cap plasmolysis. 7-day old transgenic plants were incubated with 1 M KNO_3_. (**B**) Plasmalemma was stained with 17 μg/mL CellMask™ Orange Plasma membrane Stain (orange fluorescence). (**C**) Nucleus was stained with 1 μg/mL DAPI (blue fluorescence). Fluorescence of Pt-GFP was registered at λex 488 nm, λem 505–525 nm; fluorescence of the CellMask ™ Orange, at λex 543 nm, λem 565–615; fluorescence of the DAPI, at λex 405 nm, λem 415–455 nm. Bars, 20 μm. Arrow-end line in LSM images indicates the direction along which the fluorescence profile is estimated.

For determination intracellular pH, it is important to know the location of the pH reporter protein. To make sure that the Pt-GFP is localized in the cytosol of cells and is absent in the vacuole and apoplast, the cap plasmolysis was induced in root cells. The obtained images of plasmolyzed cells show clear boundaries of the fluorescent mesoplasm, while no fluorescence is observed in the cell vacuoles (Figure 2A). The use of CellMask™ Orange fluorescent dye for plasma membrane (Invitrogen, Waltham, MA, USA) and colocalization analysis of the signals of this dye and the Pt-GFP sensor showed that the Pt-GFP is not located in the cell membrane (Figure 2B). Cell staining with DAPI nuclear dye (Bio-Rad, Hercules, CA, USA) and colocalization analysis of fluorescent signals revealed that pH probe Pt-GFP is also found in the cell nuclei (Figure 2C). 

### 2.2. Identification of Different Zones of Root Cells

**Figure 3:**
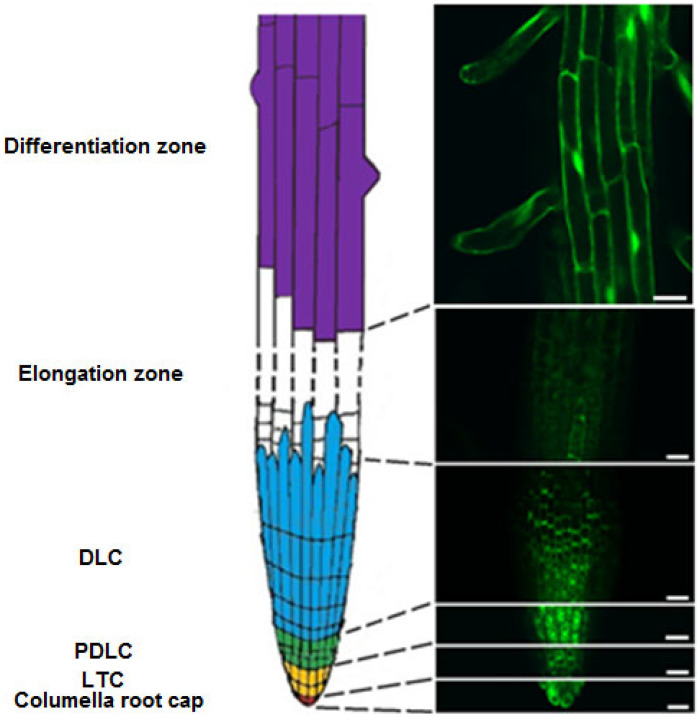
Organization of superficial cells of *Arabidopsis* root. (**Left**) layout of regions of superficial root cells. (**Right**) LSM-images of regions of superficial root cells. Fluorescent images (λem 505–525 nm) with excitation at λex 405 nm, λex 488 nm and images with merged fluorescent channels are represented. Scale bar, 20 μm. LTC—lateral root cap tip cells; PDLC—pure differentiated lateral root cap cells; DLC -differentiated lateral root cap cells.

When analyzing the obtained images of the root of *Arabidopsis* expressing green fluorescent protein, we were identified several zones of root: a differentiation zone, an elongation zone and a root cap. The differentiation zone is characterized by very long epidermal cells (up to 200 μm), localization of cytoplasm along the cell periphery and the presence of root hairs (Figure 3 and Figure S1).

On the different stages of development, cells of the elongation zone have a large number of small vacuoles or 1–3 large fused vacuoles (Figure 3 and Figure S2). The range of cell size of the elongation zone is from 8 µm to 100 µm. In contrast, to the well fluorescent cells of the differentiation zone, the cells of the elongation zone of *Arabidopsis* root had a very low fluorescent signal, which made it impossible to further study the pH.

When analyzing the obtained LSM-images of the lateral root cap cells (LRC) (Figure 3), we identified several areas of cells that are very different from each other: lateral root cap tip cells (LTC); pure differentiated lateral root cap cells (PDLC); differentiated lateral root cap cells (DLC). These areas of cells have significant differences in morphology, length, position of the nucleus, and degree of vacuolization. LTCs are located at the tip of the root around the cells of the columella (Figure S3C). They have 1–2 large mature vacuoles, and the nucleus can be located at the center of the cell or be pressed to the cell periphery. The size of LTC cells is 15–31 µm. PDLCs are represented by poorly differentiated cells of the smallest size (8–19 μm) with a large number of vacuoles and a nucleus in the center of the cell (Figure S3B). PDLCs are between LTC and DLC. DLCs are located distal to PDLCs. They have the longest cell length (up to 80 µm) among the lateral cells of the cap, 1–3 large vacuoles, the nucleus of these cells can be pressed to the plasmalemma (Figure S3A). DLC mostly protect cells of the elongation zone of the root. 

Columella cells are in a central position at the tip of the root cap in cross-section. The length of columella cells is 17–25 µm according to our data (Figure 3 and Figure S4). In the cells of the columella, there are 1–2 large vacuoles.

### 2.3. Influence of NaCl on the Length of the Root, Root Zones and Root Cells

**Figure 4:**
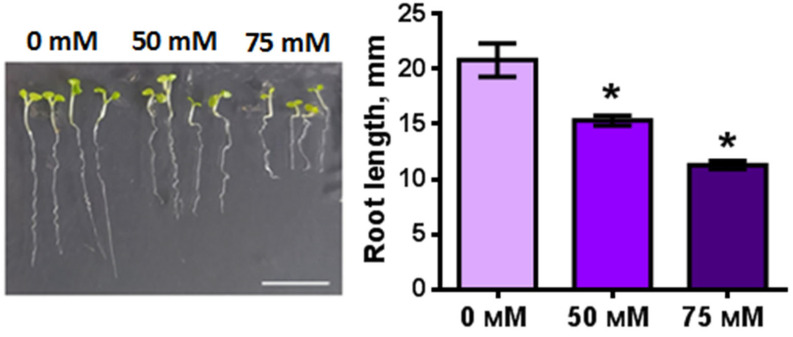
The root length of *Arabidopsis* transgenic plants grown on MS medium with 0 mM, 50 mM and 75 mM NaCl for 7 days (*n* = 15). Scale bar, 10 mm. Values are mean ± SEM. *, statistically significant difference between treatment options (ANOVA, Tukey’s test, *p* < 0.05).

The length of the root of 7-day-old *Arabidopsis* plants grown on MS nutrient medium was about 21 mm (Figure 4). Under conditions of excess NaCl (50 mM, 75 mM), the root length of *Arabidopsis* seedlings decreased on average by 5 and 10 mm, respectively (*p* < 0.05).

**Figure 5:**
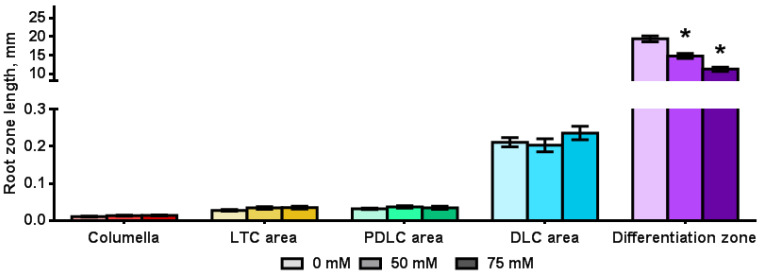
The root zone length of *Arabidopsis* transgenic plants grown on MS medium with NaCl for 7 days (*n* = 15). LTC—lateral root cap tip cells; PDLC—pure differentiated lateral root cap cells; DLC—differentiated lateral root cap cells. Values are mean ± SEM. *, statistically significant difference between treatment options (ANOVA, Tukey’s test *p* < 0.05).

We estimated the influence of salinity on the length of root zones using LSM-images of roots of transgenic *Arabidopsis* plants. The average length of the columella, the LTC area, the PDLC area, the DLC area and differentiation zone of 7-day-old plants grown on the MS was 0.011 mm, 0.027 mm, 0.032 mm, 0.211 mm and 19.396 mm, respectively (Figure 5). The presence of NaCl in the nutrient medium influenced only the length of the differentiation zone, which decreased by 4.6 and 8.2 mm under the presence of 50 and 75 mM NaCl in the medium, respectively (Figure 5). Therefore, a decrease of the length of the root of *Arabidopsis* plants under salinity occurs due to a decrease in the length of the differentiation zone. 

**Figure 6:**
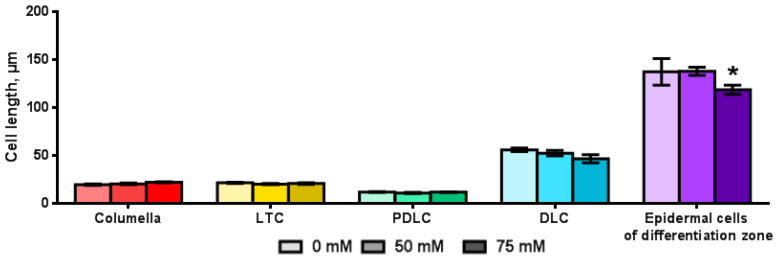
The root cells length of *Arabidopsis* transgenic plants grown on MS medium with NaCl for 7 days (*n* = 10–55). LTC—lateral root cap tip cells; PDLC—pure differentiated lateral root cap cells; DLC—differentiated lateral root cap cells. Values are mean ± SEM. *, statistically significant difference between treatment options (ANOVA, Tukey’s test, *p* < 0.05).

The length of all root cap cells did not change significantly under saline conditions (Figure 6). Only for DLC, there was a tendency of a decrease in the cell length with an increase in the salt concentration in the nutrient medium. The significant influence of NaCl on the cell length was found only for the epidermal cells of the differentiation zone. The mean of length of the epidermal cells of the differentiation zone in the presence of 75 mm NaCl decreased by about 19 μm. 

### 2.4. Study of Effect of NaCl on pH of Arabidopsis Root Cells

We studied cytosolic pH of various cells of *Arabidopsis* root using ratiometric pH indicator Pt-GFP. Both absorption wavelengths of Pt-GFP (at 405 and 488 nm) have expressed fluorescence peak at low pH; alkalization leads to a smoothing of the fluorescence peak at λex 405 nm and an increase of the fluorescence peak λex 488 nm. Therefore, we can determine cytosolic pH using fluorescence excitation ratios F488/F405, that is, the ratio of fluorescence intensity of the unprotonated and protonated forms of Pt-GFP.

**Figure 7:**
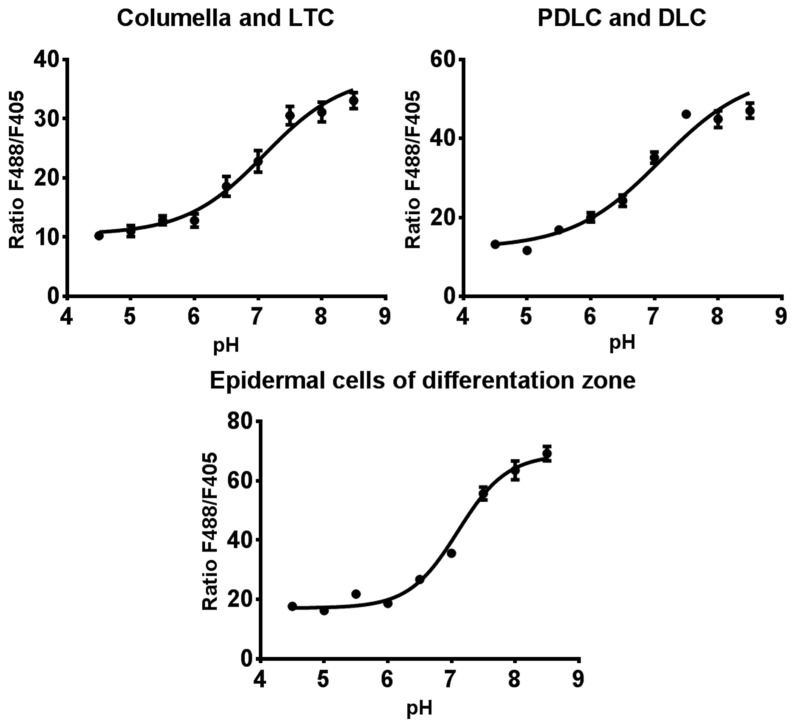
In vivo calibration of the Pt-GFP of the various regions of root cap and the epidermal cells of differentiation zone of transgenic *Arabidopsis* plants *(n* = 15–80). F488/F405 is the ratio of the fluorescence intensity of the Pt-GFP sensor when excited at wavelengths of 488 nm and 405 nm, with a registration of fluorescence at 505–525 nm. 7-days-old transgenic *Arabidopsis* plants were incubated in a solution with an appropriate pH value in the presence of the CCCP protonophore. Values are mean ± SEM.

The cells of various zones of *Arabidopsis* root have functional differences, which can be associated with the physical and chemical properties of the cytosol. These differences may affect the fluorescent signal of the pH indicator Pt-GFP. For this reason, we obtained dependencies of fluorescence excitation ratios F488/F405 of ratiometric Pt-GFP on pH for various regions of root (Figure 7). The range of minimum and maximum values of the F488/F405 ratio differed in various cell types. The minimum values of the ratio F488/F405 were from 11 to 16, and the maximum values were 34–64.

**Figure 8:**
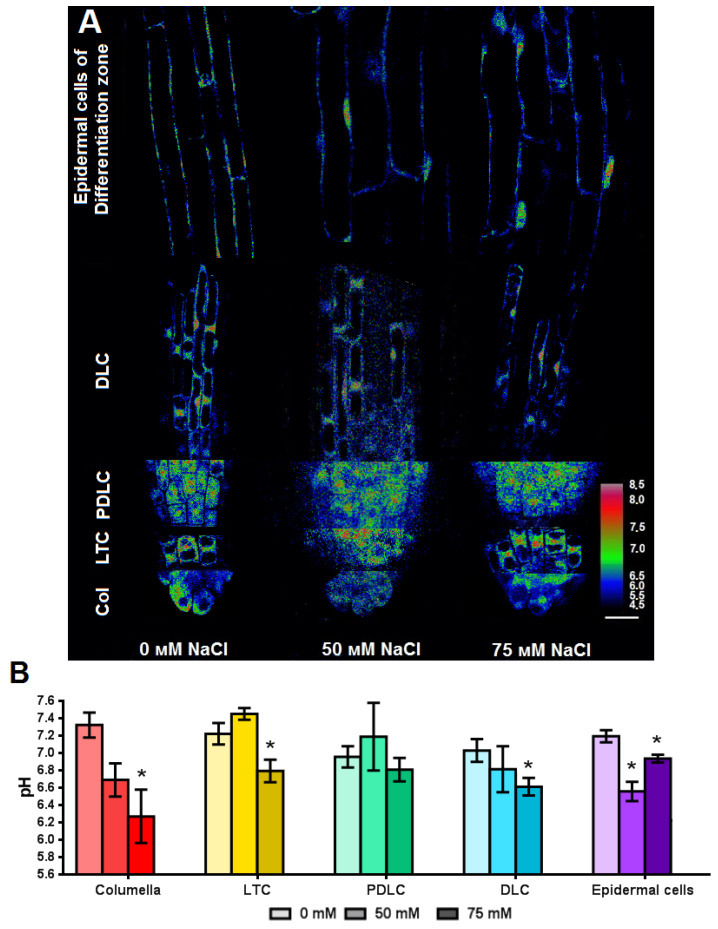
Effect of NaCl in growth medium on cytosolic pH of superficial root cells of transgenic *Arabidopsis* plants grown on MS medium with NaCl for 7 days. LTC—lateral root cap tip cells; PDLC—pure differentiated lateral root cap cells; DLC—differentiated lateral root cap cells. (**A**) Pseudocolored ratio image of Pt-GFP after a pixel-by-pixel calculation. The images were obtained by dividing the fluorescence image λem 505–525 nm, λex 488 nm, by the fluorescence image λem 505–525 nm, λex 405 nm, taking the background into account. Bars, 20 μm. (**B**) Calculated cytosolic pH level (*n* = 10–110). Values are mean ± SEM. *, statistically significant difference between treatment options (ANOVA, Tukey’s test, *p* < 0.05).

We determine the absolute pH values of the cell cytosol of each area or zone of *Arabidopsis* root using the obtained dependencies of fluorescence excitation ratios F488/F405 of ratiometric Pt-GFP on pH. The cytosol of PDLC and DLC has a slightly more acidic (pH 7.0), compared with the cytosol of columella cells, LTC and the epidermal cells of the differentiation zone—7.2–7.3 (Figure 8). Chronic salinity differently affects the cytosol pH of the surficial cells of the root. We did not find any change in the cytosolic pH of PDLC. We observed a significant acidification of the cytosolic pH by 1 unit in columella cells at the concentration of 75 mM NaCl in the nutrient medium. LTC and DLC were also only sensitive to the concentration of 75 mM NaCl, and the pH of their cytosol decreased by 0.4 units. Both concentrations (50 mM NaCl and 75 mM NaCl) significantly reduced cytosolic pH only in the epidermal cells of the differentiation zone by 0.6 and 0.4 units, respectively. Thus, the cells of the columella and epidermal cells of the differentiation zone of *Arabidopsis* root were more sensitive to salinity than LRC.

## 3. Discussion

Plant roots perform important functions such as the absorption of water and mineral elements, the synthesis and transport of physiologically active substances and responses to environmental factors. There are often identified the differentiation zone, the elongation zone, the transition zone, the meristematic zone and the root cap in the roots [34,35]. The epidermal cells of the differentiation zone and various superficial cells of the root cap have direct contact with the soil solution. The root cap surrounds and protects the meristematic stem cells and participates in modulating root growth direction and responding to various factors, including soil salinity [36,37]. In the root cap of *Arabidopsis*, two groups of cells can be distinguished; the central columella root cap and the peripheral lateral root cap [34,38,39]. In *Arabidopsis* plants cultivated on agar nutrient medium, we identified three types of LRC; differentiated lateral root cap cells (DLC) located in the proximal part of the cap and in contact with protodermal cells (future epidermal cells), pure differentiated lateral root cap cells (PDLC) located in the distal part of the cap and in contact with the columella, and nonsloughed off layer of lateral root cap tip cells (LTC). The root cap cells are eliminated by the PCD in this case, preliminary acidification of the cytosol occurs [33,37].

The primary function of the roots is the absorb water and the exchange of various compounds between the soil and the plant that is, primarily, provided by epidermal cells of the differentiation zone. Solute uptake and release by plant cells are frequently energized by the electrochemical potential difference for H^+^ or proton motive force (PMF) on plasma membrane, which is the result of a stable pH difference between the apoplast and the cytosol [18,26,40]. The antiport and symport of ions with protons are provided by PMF [41,42]. PMF is involved in the absorption of ions and substances and their removal, for example, during salinity [18,43].

The absorption capacity of the root is determined by the area of the absorption surface, which is determined primarily by the length of the root. Many factors, including salinity, affect the size of roots [44,45,46]. We have shown that the root length of 7-day-old *Arabidopsis* seedlings decreases by two times, with an increase of NaCl concentration (from 0, 50 to 75 mM). This decrease in length of root was associated with a reduction in the length of the root differentiation zone, while the average length of epidermal cells of the differentiation zone decreased only under 75 mM NaCl by 14%. A tendency to a decrease in the DLC length was also observed, which may be associated with the origin of the DLC and epidermal cells of the differentiation zone from the same initial cells of the root apical meristem [39]. We suggest that the reason for the decrease in root length under salinity (Figure 6) is the inhibition of the activity of meristem cells and the transition zone, as shown in some reports [35,36,47].

One of the regulators of cell division and elongation is indole-3-acetic acid (IAA). The transport of IAA changes in the root cap under salinity, particularly the internalization of PIN2 carriers and disruption of the exit the PIN4 to plasmalemma leads to inhibition of the exit of IAA to the elongation zone [36,44,48]. In addition, accelerated deposition of lignin and other components of the cell wall reduces the ability of cells to elongate under salinity [36]. In general, the high content of Na^+^ and Cl^−^ in the cytosol inhibits the activity of many enzymes (Na^+^) and disrupts the absorption of other anions through nonselective anion channels (Cl^−^), mostly NO_3_**^−^** and SO_4_^2−^, [11], which, ultimately, leads to inhibition of root growth, root length and reduction of the differentiation zone.

In this study, we showed that the cytosolic pH of cells in various root areas differ slightly; 7.3 for columella cells; 7.2 for LTC cells; 7.0 for PDLC and DLC cells; 7.2 for epidermal cells of the differentiation zone. Previously, other researchers determined the pH of the cytosol of plant cells under normal conditions in the range of 6.9–8.0 [15,19,20,23,25,26,32,49]. Schulte et al. [21] showed that the cells of the elongation zone near the root tip have some differences in cytoplasmic pH (7.4–7.6) compared to the zone of root hairs near the hypocotyl (7.2–7.3). Martinière et al. [26] reported small differences in cytosol pH of the cells of the epidermis, cortex, endodermis and stele of the differentiation zone of *Arabidopsis* roots. Thus, the pH values of the cytosol of various superficial corresponded to the results of other studies. The maintenance of a pH in the plant cell is achieved by the work of P-type H^+^-ATPase of the plasmalemma H^+^-pyrophosphatases and V-type H^+^-ATPase of the tonoplast and a biochemical pH-stat [18,41,50]. The biochemical pH-stat combines the work of several enzymes that metabolize organic acids. The various H^+^-ATPase, H^+^-pyrophosphatases and enzymes of biochemical pH stat can have different expression, amount and activity in various root cells [12,51,52,53], which may be responsible for the differences in cytosolic pH values of various cells of *Arabidopsis* root.

The superficial cells of *Arabidopsis* root were sensitive to the salinity. However, a response to a concentration of 50 mM NaCl with decrease in cytosolic pH was found only in epidermal cells of the differentiation zone. Most types of superficial cells (columella, LTC, DLC, epidermal cells of the differentiation zone) had acidification of the cytosol under 75 mM NaCl in the nutrient medium (Figure 8). Acidification of the cytosol under salinity was also shown for root cells of *Arabidopsis* (zone not indicated) [30] and cells of the hairy zone near the hypocotyl of *Arabidopsis* root [21]. On the other hand, according to our data, the cytosolic pH of PDLC did not change. Thus, there is cell-type-specific cytosolic pH response of the superficial cells of *Arabidopsis* seedlings root to chronic salinity, while the most pronounced changes in cytosolic pH we recorded in the epidermal cells of the differentiation zone and columella, which are involved in the processes of absorption of substances and tropisms.

The reasons for the change in cytosolic pH may be related to the feature of the regulation of the Na^+^ concentration in plant cells. Extrusion of excess sodium out of the cytosol is carried out by Na^+^/H^+^ antiporters of the plasmalemma (SOS1, CHX13) [11,18,54,55] and the tonoplast (NHX1-6) [56], which remove Na^+^ ions from the cytosol and introduce H^+^ into the cytoplasm, which leads to acidification of the cytosol. It should be noted that sodium transporters, for example, NHX, are differently expressed in various root cells; particularly, a high content of the AtNHX1 protein was shown in the differentiation zone, while AtNHX1 was not found in the root tip [57]. In addition, Na^+^ influx to the cell leads to inhibition of H^+^-ATPase activity and acidification of the cytosol [11]. On the other hand, there are data indicating that salinity increases the activity of PM H^+^-ATPase [44,58,59], which is necessary to the active work of Na^+^/H^+^ antiporters [60].

Probably, the aforementioned proteins in the various superficial cells are presented and regulated in different ways and have different sensitivity to the salt concentration, which is the reason for the cell-type-specific cytosolic pH response that we found in these cells under normal conditions and salinity.

In general, in the performed study, heterogeneity of the cytosolic pH value is demonstrated in the superficial cells of *Arabidopsis* root under normal conditions and the heterogeneity of cytosolic pH response under chronic salinity. The cells of the columella and epidermis of the differentiation zone, which participate in perception of external signals and absorption of nutrients, have a more alkaline pH level of the cytoplasm, which leads to a large electrochemical gradient of protons on the plasmolemma, which is an energy source for both ion transport and signaling of external influences. In these cells, we found the greatest acidification of the cytosol in the presence of NaCl, which may have a signaling function and may be a result of excess of Na^+^ outside of cell because PMF is necessary for extrusion of Na^+^ from cell that leads to acidification. The decrease in the length of the differentiation zone under salinity indicates a disruption in the activity of the meristem and elongation zones, which could be directly exposed to salt stress or, probably, could receive signals from the columella. However, this issue requires further detailed study.

## 4. Materials and Methods

### 4.1. Plant Material and Growth Conditions

Studies were carried out on plants of *Arabidopsis thaliana* (L.) Heynh. ecotype Columbia (Col-0) and transgenic line PtGFP_kn expressing pH-reporter protein Pt-GFP in the cytosol (Notthingham Arabidopsis Stock Centre, University of Nottingham, Sutton Bonington Campus, UK) in the Col-0 background.

The fluorescent pH indicator Pt-GFP from sea pen *Ptilosarcus gurneyi* Gray has wide optimal pH-range of sensitivity (4.5–8.5) [21]. Pt-GFP is readily expressed in all cells of *Arabidopsis* without additional modifications [21]. Pt-GFP is a fluorescent ratiometric indicator because it has two excitation peaks at 390 and 502 nm [21,61]. This pH probe does not have chloride sensitivity [21].

Plants were grown at 25 °C (80 µmol m^−2^ s^−1^ irradiance; 16-h daylength) on Murashige and Skoog medium (MS) [62] containing 30 g L^−1^ (*w*/*v*) sucrose and 7 g L^−1^ (*w*/*v*) agar and 50 mM or 75 mM NaCl. Control plants were grown under the same conditions on the same medium without NaCl. Seven-d-old seedlings were used for investigation.

### 4.2. Confocal Microscopy of Root Cells of Arabidopsis

LSM images and fluorescent spectra of different root cells of control and transgenic *Arabidopsis* plants were obtained by an LSM710 confocal laser scanning microscope (Carl Zeiss, Jena, Germany) with a EC Plan-Neofluar 20×/0.5 M27 or EC Plan-Neofluar 10×/0.3. g objectives. Pt-GFP was excited by 30 mV diode laser at 405 nm and 35 mV argon laser at 488 nm and detected between 505 nm and 525 nm. The power of the light flux of the two used lasers was aligned using a Thorlabs PM100A power meter with an S121C sensor (Thorlabs, Bergkirchen, Bavaria, Germany). LSM imaging was carried out at a laser power of 129 μW and a detector sensitivity of 540 V.

For LSM analysis in vivo, a thin plate of the agar medium with root of 7-day-old *Arabidopsis* seedling was cut out and clamped between two coverslips. The shoot of *Arabidopsis* seedling was on the surface of the coverslip.

For determination of the localization of Pt-GFP in the cell, plasmolysis and staining of plasma membranes and nucleus were performed. To induce cell plasmolysis, plants were incubated in 1 M KNO_3_ for 40 min. Then, LSM images were obtained. The plasma membrane was stained with CellMask™ Orange Plasma membrane Stain fluorescent dye (Invitrogen, Waltham, MA, USA). For staining *Arabidopsis* roots were placed in a fluorescence dye solution of 16.7 μg/mL in phosphate buffer (pH 7.4), in which and LSM images were immediately obtained (λex 543 nm, λem 565–615 nm). To determine the localization of the nucleus, *Arabidopsis* roots were stained with DAPI dye (Bio-Rad, Hercules, CA, USA) (1 mg/mL in phosphate-buffered saline (pH 7.4). Plants were incubated in this solution for 20 min, and then LSM images were received (λex 405 nm, λem 416–453 nm).

### 4.3. Assessment of Morphological Parameters of Root

To determine the effect of NaCl on the length of plant roots *Arabidopsis* plants were cultivated on MS media supplemented 0, 50 mM or 75 mM NaCl for a week. Then, plants were photographed with a millimeter ruler as a reference. The length of the roots in the obtained photographs was calculated using the ImageJ 1.40 g program [63].

The contents of the cells were well visualized because their cytosol has fluorescent protein. The length of individual root zones and the length of cells of these zones were measured on LSM images of the roots of transgenic plants. Root zones and areas of root cap were determined by cell morphology, the presence of root hairs, cell length and the degree of vacuolization. The micrometer scale for each image was set using the ZEN 2012 software (Carl Zeiss, Jena, Germany). Calculation of the length of the zones and cells of the roots of transgenic plants was carried out on LSM images using the ImageJ 1.40 g program [63].

### 4.4. In Vivo Calibration of Pt-GFP Signal and Ratiometric Analysis in Arabidopsis Plants

*Arabidopsis* roots were imaged using ZEN 2011 SP4 (black) 11.0 software. Pt-GFP fluorescence intensity in the cell cytosol at λex 405 nm (F405) and λex 488 nm (F488) were evaluated on the regions of interest (ROI) of LSM images of cells. The background values were subtracted from F405 and F488. The autofluorescence intensity of the cell cytosol of plants of *Arabidopsis* ecotype Columbia at λex 488 nm (Fbackground488) and λex 405 nm (Fbackground405) were taken as the background after that the values of the ratio of the fluorescence intensity of the pH probe (F488-Fbackground488)/(F405-Fbackground405) in the cells of the control plants and the plants grown under saline conditions was determined.

The dependence of the ratio (F488-Fbackground488)/(F405-Fbackground405) of *Arabidopsis* root cells on pH was obtained for calculating of the absolute cytosolic pH values of the cells. For this, whole 7-day-old transgenic *Arabidopsis* seedlings were incubated in buffers of different adjusted pH (4.5 to 8.5) overnight. Buffer solutions had the following composition: c pH 4.5, 5.0, 5.5, 6.0 (40 mM sodium citrate, 40 mM MES, 40 mM MOPS), 6.5, 7.0 (40 mM sodium citrate, 40 mM MES, 40 mM MOPS, 40 mM TRIS), 7.5 (40 mM MOPS, 40 mM TRIS), 8.0, 8.5 (80 mM TRIS). The base of all buffers was a standard solution (1 mM NaCl, 0.1 mM KCl, 0.1 mM CaCl_2_). Buffer solutions contained 250 µM of the protonophore carbonyl cyanide 3-chlorophenylhydrazone (CCCP) for balancing of the pH inside and outside the cell. Before the LSM analysis, the pH of all buffer solutions with incubated plants was re-evaluated using a pH meter. For LSM microscopy, the plants were placed on a coverslip in 0.3 mL of the appropriate buffer solution. Next, LSM images of cells of different root zones were obtained.

The intensity of autofluorescence or Pt-GFP fluorescence at λex 488 and λex 405 nm were evaluated and the ratios (F488-Fbackground488)/(F405-Fbackground405) were calculated on the obtained images of control and transgenic *Arabidopsis* plants. The curves of dependence (F488-Fbackground488)/(F405-Fbackground405) on pH were built according to the obtained data. These curves were approximated by sigmoid, and equations were obtained using the software GraphPad Prism 6.01 (GraphPad Software Inc., San Diego, CA, USA). The resulting equations were used to determine the cytosolic pH of *Arabidopsis* plants grown with NaCl.

### 4.5. Statistical Data Analysis

To determine the effect of salinity on morphometric parameters, 15 plants of each group were used. To determine the effect of salinity on cytosolic pH, 6–9 plants of each group were used. We carried statistical processing of the results out using MS Excel (Microsoft Corporation, Redmond, WA, USA) and GraphPad Prism 6.01 software. Data were analyzed using one-way analysis of variance (ANOVA) followed by Tukey’s test. Data are represented as mean ± standard error of mean (SEM).

## 5. Conclusions

We have shown a gradient of cytosolic pH values in various superficial types of *Arabidopsis* root cells. We did not find changes in the length of the root cap cells, while there was a decrease in the length of the differentiation zone (50, 75 mM NaCl) and the size of the epidermal cells of the differentiation zone (75 mM NaCl). According to our data, the most of LRCs have neutral cytosol pH values, while the cells of the columella and epidermis of the differentiation zone have weakly alkaline cytosol pH values. Along with the anatomical differences in the response of superficial cells to chronic salinity, they have a heterogeneous response of cytosolic pH to salinity.

## Data Availability

Not applicable.

## Supplementary Materials

The following supporting information can be downloaded at: https://www.mdpi.com/article/10.3390/plants11243532/s1, Figure S1. LSM-image of epidermal cells of differentiation zone of Arabidopsis root (λex 488 nm, λem 500–525 nm). Scale bar, 20 μm. Figure S2. LSM-images of elongation zone of Arabidopsis root (λex 488 nm, λem 500–525 nm). V—vacuoles. Scale bars, 20 μm. Figure S3. LSM-images of lateral root cap cells of Arabidopsis root (λex 488 nm, λem 500–525 nm). (A) Differentiated lateral root cap cells (DLC). (B) Pure differentiated lateral root cap cells (PDLC). (C) lateral root cap tip cells (LTC). Scale bars, 20 μm. Figure S4. LSM-image of columella of Arabidopsis root (λex 488 nm, λem 500–525 nm). Scale bar, 20 μm.
