# Supplementary material for: Cell-Type-Specific Length and Cytosolic pH Response of Superficial Cells of Arabidopsis Root to Chronic Salinity
Source: Plants (Basel). 2022 Dec 15;11(24):3532. doi: 10.3390/plants11243532 (PMC9783886; doi:10.3390/plants11243532)
Supplement: Supplementary file 1 [file plants-11-03532-s001.zip › plants-2072940-supplementary.pdf]

Supplementary Materials

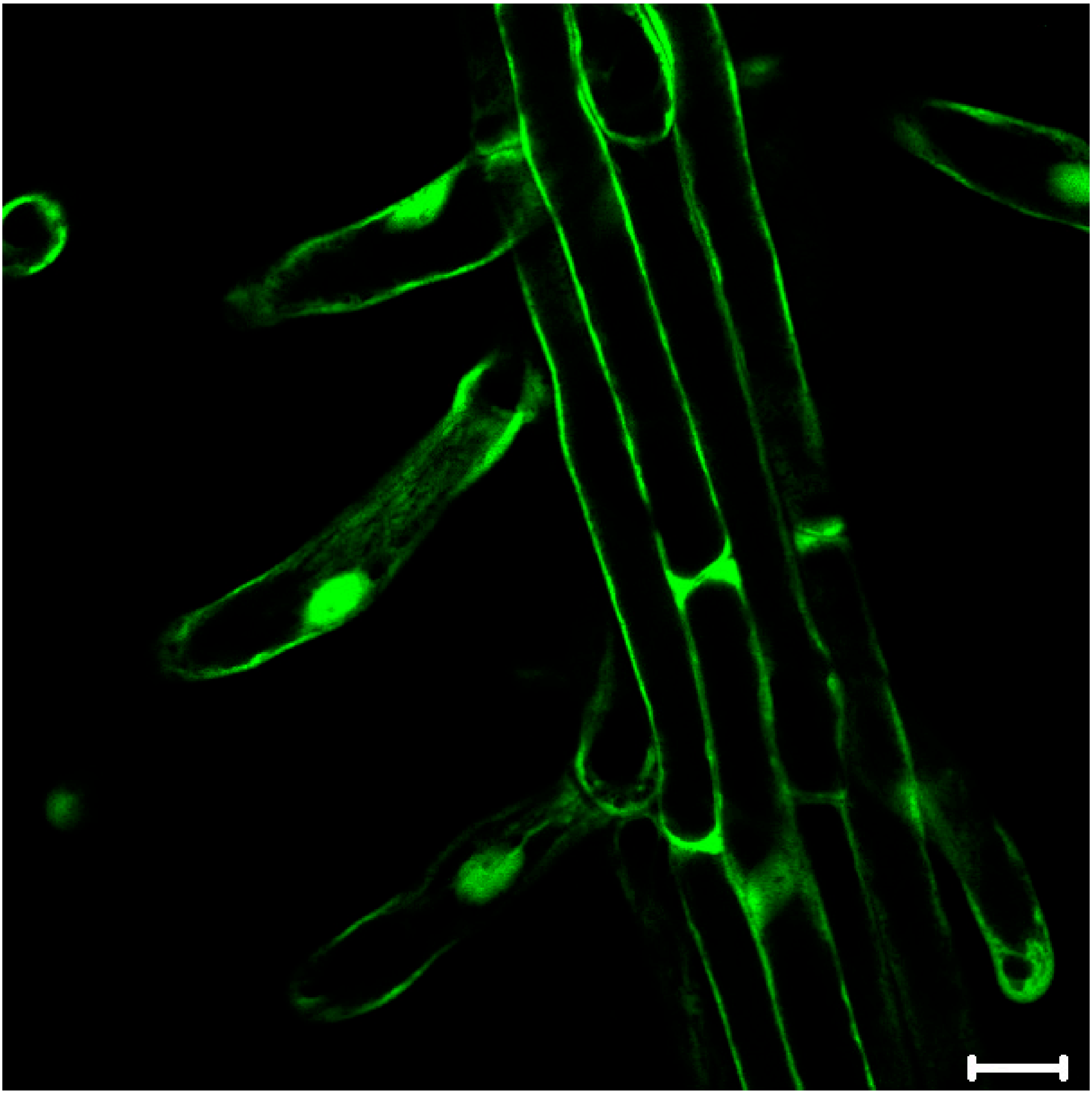

Figure S1. LSM-image of epidermal cells of differentiation zone of Arabidopsis root (λex 488 nm, λem 500–525 nm). Scale bar, 20 μm.

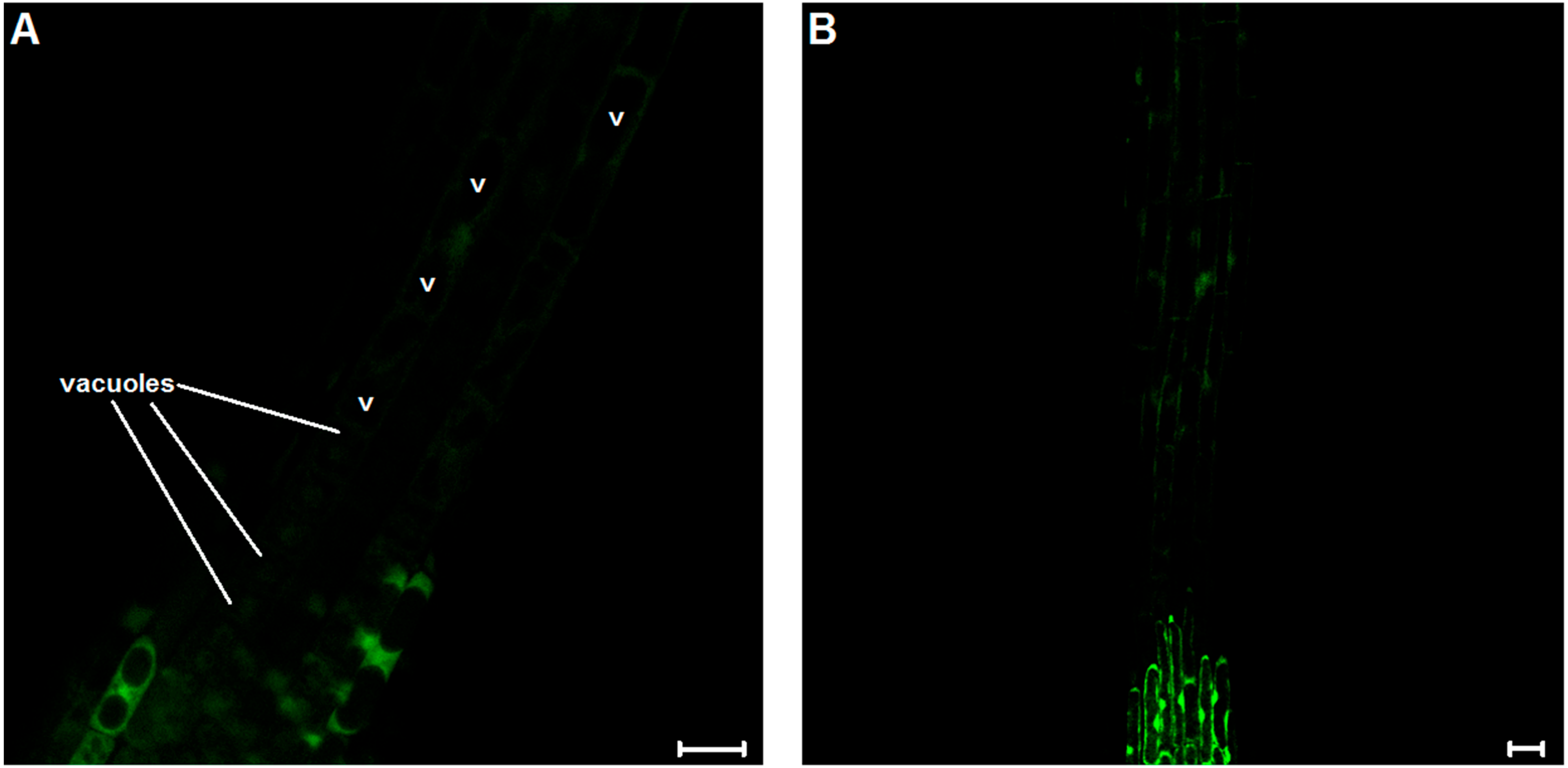

Figure S2. LSM-images of elongation zone of Arabidopsis root (λex 488 nm, λem 500–525 nm). v – vacuoles. Scale bars, 20 µm.

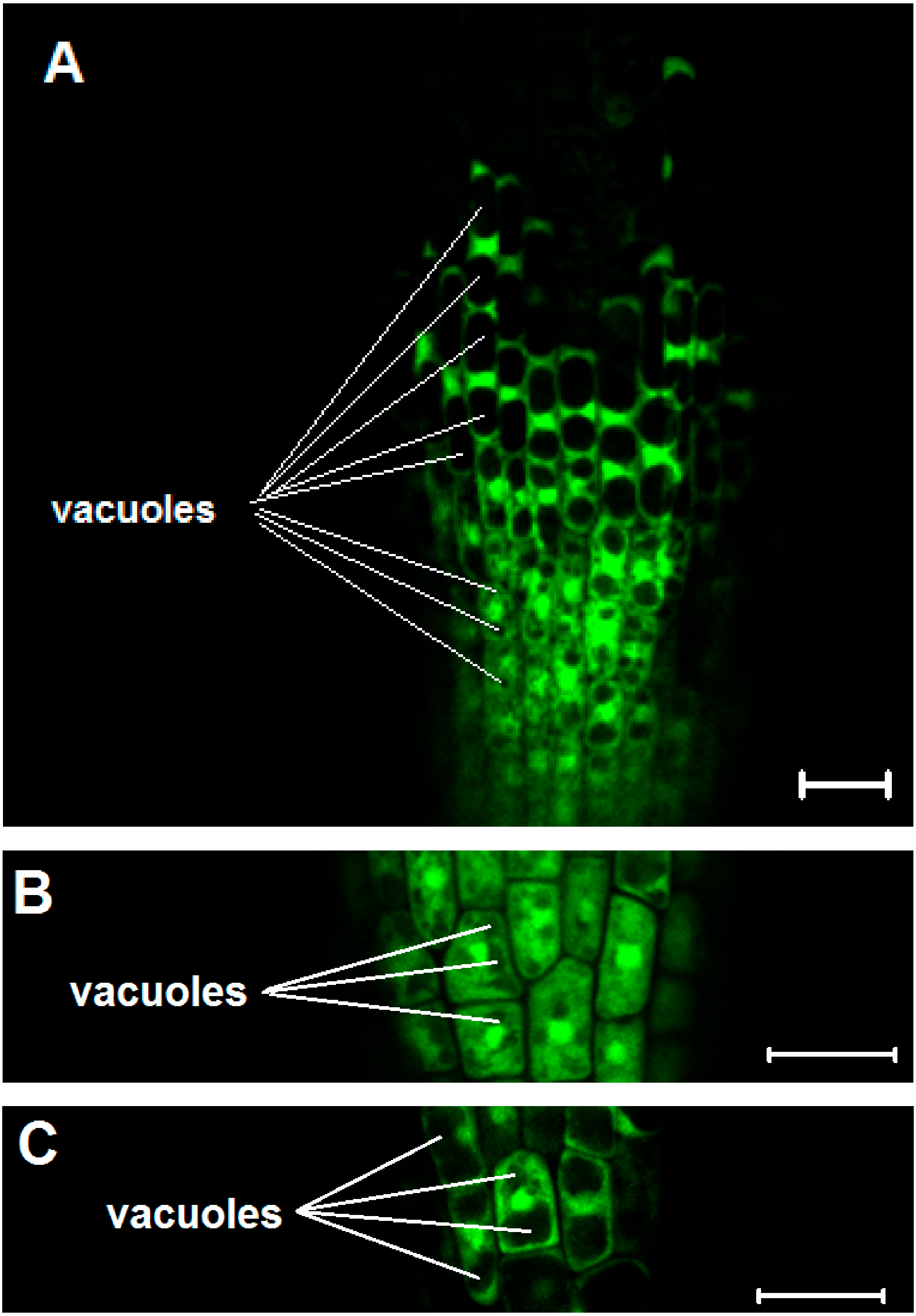

Figure S3. LSM-images of lateral root cap cells of Arabidopsis root (λex 488 nm, λem 500–525 nm). (A) Differentiated lateral root cap cells (DLC). (B) Pure differentiated lateral root cap cells (PDLC). (C) lateral root cap tip cells (LTC). Scale bars, 20 μm.

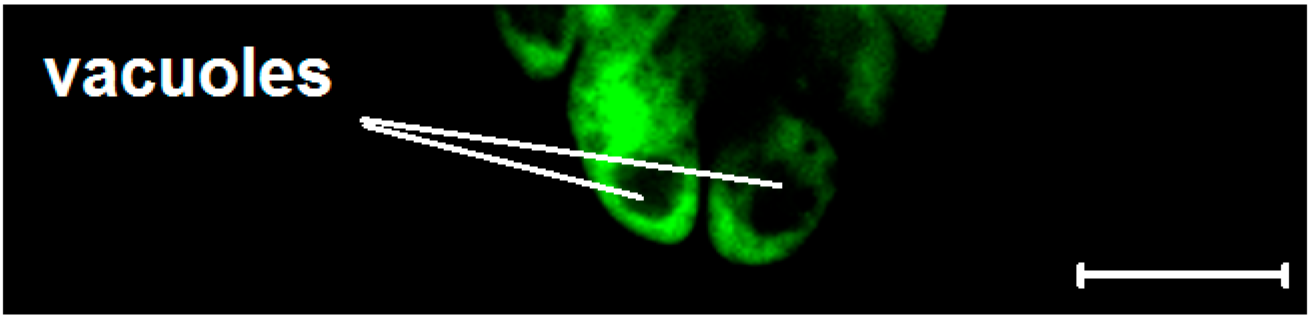

Figure S4. LSM-image of columella of Arabidopsis root (λex 488 nm, λem 500–525 nm). Scale bar, 20 μm.
